# The Wily and Courageous Red Fox: Behavioural Analysis of a Mesopredator at Resource Points Shared by an Apex Predator

**DOI:** 10.3390/ani9110907

**Published:** 2019-11-01

**Authors:** Eamonn Wooster, Arian D. Wallach, Daniel Ramp

**Affiliations:** Centre for Compassionate Conservation, University of Technology Sydney, P.O. Box 123, Ultimo, New South Wales 2007, Australia; Arian.Wallach@uts.edu.au (A.D.W.); Daniel.Ramp@uts.edu.au (D.R.)

**Keywords:** *Vulpes vulpes*, *Canis dingo*, landscape of fear, trophic cascades, mesopredator

## Abstract

**Simple Summary:**

The red fox is one of the Earth’s most widespread mammalian predators. Human globalisation has further expanded its range, so that today they are found on most continents. Despite their abundance, knowledge of fox behaviour remains limited. Most studies have observed foxes either in captivity or in their native range where both they and their predators are killed by humans. We conducted a behavioural study on foxes outside of their native range in Australia, at a unique location where all wildlife are protected. We developed an ethogram to explore fox behaviour at resource points shared with a potentially deadly apex predator, the dingo. We were surprised to find that foxes were in a confident state more often than in a cautious state, even leaving territorial markings over those of dingoes. One possible explanation for the confidence of foxes is that the social stability of both foxes and dingoes makes their world more predictable.

**Abstract:**

The red fox (*Vulpes vulpes*) is a widespread and ecologically significant terrestrial mesopredator, that has expanded its range with human globalisation. Despite this, we know relatively little about their behaviour under the wide range of ecological conditions they experience, particularly how they navigate the risk of encounters with apex predators. We conducted the first ethological study of foxes outside their historic native range, in Australia, where both the foxes and their main predator were protected from human hunting. Using remote camera traps, we recorded foxes visiting key resource points regularly utilised by territorial dingoes (*Canis dingo*), their local apex predator, in the Painted Desert, South Australia. We constructed an ethogram sensitive to a range of behaviours and attitudes. Since foxes are suppressed by dingoes, we expected that the foxes would primarily be in a cautious state. In contrast, we found that foxes were in a confident state most of the time. Where human hunting is absent, social stability of predators may increase predictability and therefore decrease fear.

## 1. Introduction

“*Look at him. His coat is russet with sufficient gold in it to make him glow. He has just enough of a ruff to please a very young lion and enough tail to be the pleasure of any animal that grows a tail. His snout is a bit pinched-looking and would be mean if there wasn’t so much pride in his carriage—he walks the Australian earth as though it was a carpet especially laid for him*”.

(Rolls 1969)

The red fox (*Vulpes vulpes*) is one of the most widespread of all carnivores, having populations on all continents except Antarctica and South America [1]. They inhabit a wide range of habitats, including tundras, temperate woodlands, coasts, and deserts. Foxes have adapted to, and benefited from, the ecological changes of the Anthropocene, exploiting anthropogenic resources [2] and experiencing release from predation through the extirpation of apex predators in both urban and agricultural landscapes [3]. Foxes have also significantly expanded their historic range through introductions by humans, establishing populations in nine new countries over the last 170 years [4]. Studying the behaviour of animals outside of their historical ranges provides a unique opportunity to explore how species and individuals adapt to the challenges and opportunities of new environments.

One population that has flourished due to human-assisted migration is in Australia. Foxes were first introduced to Australia in the 1830s, brought to Victoria for hunting. Within a century they had expanded their range throughout much of the continent [5]. Bounty programs and “pest” status were first established in the 1890s, both of which continue today. Foxes are routinely shot, poisoned, trapped, and gassed, everywhere from national parks to farms and urban parklands. These lethal programs exist alongside a similar campaign against dingoes (*Canis dingo*), Australia’s only remaining mammalian apex predator. This has left foxes in a state of “mesopredator release”, removed from top-down pressure [6]. Hence, although foxes are heavily targeted by control programs, these efforts have not led to local or functional extirpation.

Apex predators limit the densities and spatial distribution of smaller predators, through competition, predation, and intraguild competitive killing [7]. The ecology and behaviour of foxes, in both their historic and introduced ranges is actively shaped by predation and interference by apex predators [3,8]. Within their historic native range, foxes are suppressed by coyotes in North America (*Canis latrans*) [9] and wolves in Europe (*Canis lupus*) [10], while in Australia they are suppressed by dingoes [11]. In response to the presence of apex predators, foxes are known to alter spatio-temporal activity patterns and increase vigilance behaviour, helping them to detect and avoid risky encounters [12,13]. This creates a “landscape of fear”, represented by “peaks” (high risk) and “valleys” (low risk) [14]. In North America, foxes have been shown to exploit urban areas to avoid coyotes [9], while in the Australian desert, foxes avoid water sources where dingo activity is concentrated [15].

A review of ethological studies revealed that most research on wild foxes has occurred where apex predators were absent (80%) and where foxes and their predators were subjected to lethal control (83%) (Table 1). Only two studies were conducted where both foxes and their predators were protected [12,16], both within the fox’s historic range. A further eight studies were conducted in the wild that did not report whether apex predators were present, protected, or killed; three studies were conducted in captivity; while the rest were conducted without apex predators present. To the best of our knowledge, no study has been concerned solely with understanding fox behaviour outside their historic range. Although there has been much research on foxes in Australia, most of it has been concerned with how to suppress their populations [17,18]. Our interest, therefore, was in developing a suitable ethogram of fox behaviour and then implementing that ethogram to study wild fox behaviour in an introduced setting without interference from human persecution of themselves or of their predators. To do this, we made use of a rare ‘predator friendly’ landscape in the Australian desert to observe fox behaviour where they are at risk from potentially deadly encounters with dingoes. Due to the high level of risk dingoes can pose to foxes, we expected that foxes would be highly cautious when accessing resource points shared with territorial dingoes.

## 2. Materials and Methods

### 2.1. Study Sites

Our study was conducted across two contiguous predator friendly properties in the Painted Desert, South Australia. The properties include a 2300 km^2^ cattle station and a 5600 km^2^ Indigenous Protected Area, utilised in part for cattle and horse grazing. Foxes are likely to have been resident in the area as early as 1940, by which point they were already present in over two-thirds of Australia [39]. The landscape is arid, with average rainfall around 160 mm annually, and is dominated by chenopod shrublands, tall Acacia woodland, and Eucalyptus species along ephemeral creeks. Reliable sources of drinking water were limited to semi-permanent rain-filled dams and permanent bores spread evenly across the landscape, approximately every 10 km. Historically, predators had been regularly poisoned, shot, and trapped across the region, but non-lethal predator friendly practices were established on both properties by 2012 [40].

### 2.2. Recording Fox Behaviour

We remotely filmed foxes at water sources, rabbit warrens, and large carcasses; resource points known to be utilised by territorial foxes and dingoes [41]. Predators are highly elusive and thus, behavioural data is difficult to obtain as direct observations are not possible. This makes camera trapping the only source of gathering such data. Camera traps were placed at water points approximately 10 km apart, however, rabbit warrens and carcasses were usually located within 3 km of the water points. As we were unable to identify individual foxes, and foxes home ranges vary between 8 – 33 km^2^ in arid environments [42], we caution that it is possible that we observed the same individuals across multiple resource points. Water sources in the arid zone are important resources for predators; for drinking, socialising, communicating, and hunting [15,43]. The highest concentration of dingo scent marking occurs at arid zone water sources, with some waters having over 100 dingo scats. Dingo scent marking concentrates in areas where they are socially stable [40]. Scent marking is an indicator of social stability in canids as it is a common method of communicating sociality and territoriality. Large carcasses are important as resource points for food and as focal points for scent marking for both foxes and dingoes [41]. Foxes readily scavenge carcasses of large prey killed by apex predators [44], and both foxes and dingoes scavenge domestic animals discarded by humans [45]. Both predators are also significant predators of rabbits (*Oryctolagus cuniculus*) [45,46] and use rabbit warrens to locate prey and for scent marking [28,41]. Rabbits warrens are burrows dug and utilised by rabbits for shelter and raising young, they are identified through tracks or scats present at their entrance. We focused this study on resource points as they are both essential parts of life within the desert for both foxes and dingoes and as they represent potential points of conflict between them. 

We monitored fox behaviour through 3 winters (June–July; 2016–2018). In 2016, we monitored 10 water points; in 2017, we monitored 18 water points (of which eight were also monitored in 2016), 17 rabbit warrens, and five carcasses; and in 2018 we monitored 10 water points (of which six were also monitored in 2017, and three were monitored over all three years), eight rabbit warrens, and four carcasses (two of which were monitored for three years). We strapped camera traps (Bushnell MKII and Browning Dark Ops Pro) to trees and posts at 30–60 cm high for 1–3 weeks set to record time-stamped 15–20 s videos, with one second delays. Cameras were hidden to the best of our ability to reduce the chance of behavioural responses being influence by the camera traps themselves. Cameras were active 24 h a day and were checked at least once a week. We set up to three cameras per water source, up to two cameras per rabbit warren, and up to two cameras per carcass, with the number varying based on the size of each resource point. We treated points independently for temporal analysis, if foxes were present on more than one camera at a single resource point within 30 min of each other, we considered them part of the same activity event [47].

### 2.3. Construction of a Fox Ethogram

**Figure 1:**
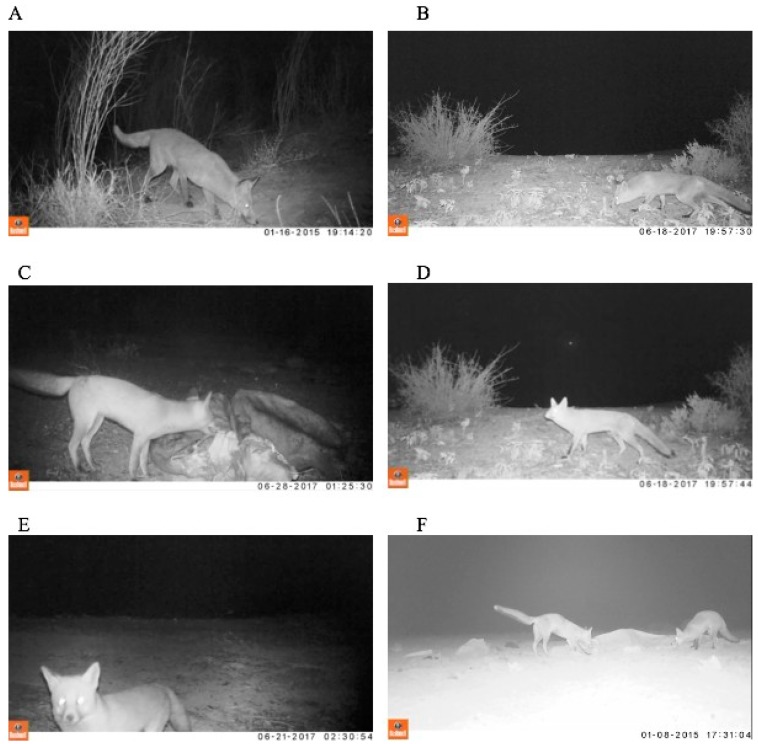
Behaviours observed in this study and used to classify fox behaviour: (**A**) confident sniffing and walking, (**B**) cautious sniffing and walking, (**C**) confident scavenging, (**D**) high vigilance, (**E**) cautious camera investigation, (**F**) social foraging. See supplementary material 1 for an example of behaviourally scored video.

We identified and described discrete fox behaviours to create an ethogram sensitive to wariness of foxes to predation while accessing resource points. We characterised fox behaviours from literature in ethology [25,48,49,50], animal personality [51], and animal welfare [52], and from assessments made by captive fox carers (Sydney Fox Rescue). The ethogram was first organised into base behaviours, describing key actions such as locomotion and foraging (Table 2). Base behaviours were classified as either state events or point events for purposes of measurement. State events were defined as continuous behaviours (e.g., locomotion) and were measured in units of time (>1 sec), while point events were defined as instantaneous behaviours (e.g., startled jump) and were measured in units of frequency (<1 sec). All base behaviours were further refined through modifiers, which were descriptive terms used to contextualise base behaviours both physically and mentally [53]. For example, ‘locomotion’ was modified by a range of both physical states, such as walking, running, jumping, or perching, and by attitudinal states, such as whether the actions were engaged in confidently or cautiously. Attitudinally modified behaviours were classified as either confident or cautious based on the foxes’ body position. Cautious behaviour is primarily categorised by the tail positioned below the height of the back, the torso positioned close to the ground, and the legs spread apart. Cautious behaviour shares body positions with vigilant behaviour. In contrast, confident behaviours were primarily classified where the foxes are observed with the tail held above or level to the back, legs are extended and positioned close together (Figure 1, Table 3, Supplementary Material Table S1).

Behavioural analysis of videos was performed using the behavioural analysis software BORIS version 4.1.4 [53]. We analysed observed behaviours for duration and/or frequency, according to the definitions in our ethogram. We calculated the proportion of time each base behaviour and modifier combination contributed to the total time of fox behaviour. Point events were analysed exclusively for their frequency of occurrence. Where more than one fox was present, behaviour was analysed separately. We tested for differences in the proportion of time allocated to each base behaviour between the surveyed resource points, and for differences in the proportion of confident and cautious behaviour within and between each resource point, using separate negative binomial regressions, one per behaviour (link function: log). All proportions were modelled as integers. In each regression, we set the proportion of time allocated in a given behaviour as the response variable. We included resource type as the predictor variable. We tested significance of the predictor using a Tukey post hoc test in R version 3.4.1 using the package emmeans [54]. Behaviours with only one attitudinal modifier (e.g., vigilance and scent marking) were removed from this analysis. Digging was also left out as it was only observed once. Negative binomial regressions were performed through the R version 3.4.1 using the package MASS [54].

We analysed fox activity patterns at each of the three resource points (i.e., water sources, rabbit warrens, and carcasses). Temporal activity patterns were compared using kernel densities, to estimate activity overlap between the resource points by calculating the area under the curve where all three temporal patterns overlapped. We did this by calculating the densities at which each temporal pattern intersected and then integrated the area where all resource points overlapped, compared to the total curve area. Finally, we recorded the frequency foxes were observed alone or in company. All analyses were performed in R version 3.4.1 [54].

## 3. Results

We identified 14 base behaviours useful for categorising activity around resource points: locomotion, sniffing, digging, vigilance, foraging, flight, investigating, frustration, salivating, head shake, play, greeting, and resting (Table 2, Figure 1). We also identified five modifiers for those behaviours: type (e.g., locomotion modified as walking or running), attitudinal (e.g., locomotion modified as cautious or confident), intensity (e.g., vigilance modified as high or low), and social (e.g., foraging modified as social or alone) (Table 3).

**Figure 2:**
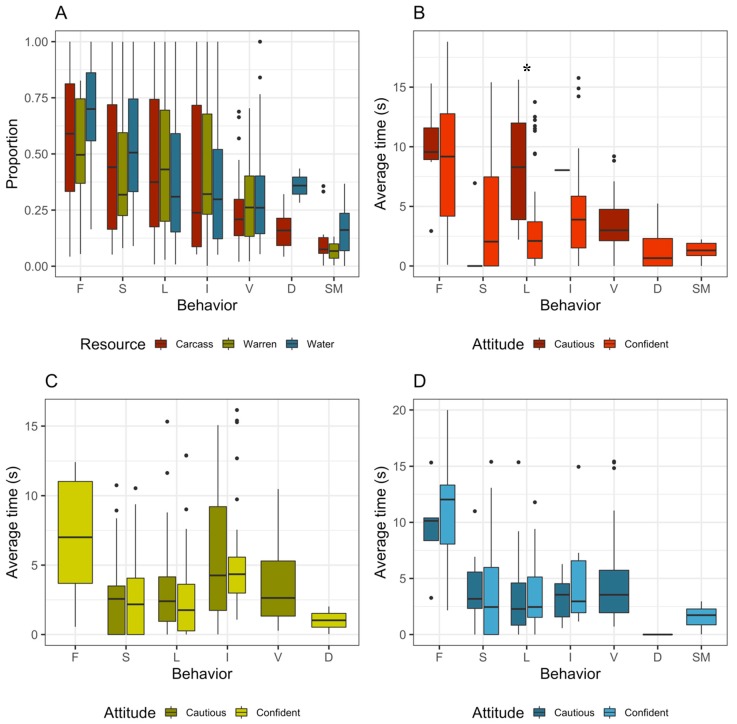
Confidence and cautiousness of red foxes at key resource points share with dingoes. Proportion of time allocated to each behaviour at each resource type (**A**). The average amount of time allocated to confident and cautious behaviours at carcasses (**B**), rabbit warrens (**C**), and water points (**D**). F = foraging, S = sniffing, L = locomotion, I = investigating, V = vigilance, D = digging, SM = scent marking. Statistical significance indicated by an asterisk.

We gathered a total of 55.33 min of fox footage (1.33 min from 2016, 42 min from 2017, and 12 min from 2018). Dingoes were present at all resource points surveyed, with evidence of scent marking by dingoes recorded at all carcasses, at 97% of water points, and at 47% of rabbit warrens. While accessing resource points, foxes spent most of their time engaged in the relevant foraging behaviour associated with that resource (e.g., scavenging at carcasses, and drinking at water points), as well as sniffing and locomoting (Figure 2). There were no major differences in behavioural activity between the three resource types. On average, foxes spent 12 s on camera, with the longest recorded at 65 s.

The average proportion of time allocated to a behavioural state was independent of the attitude of the fox (i.e., the time did not change whether the behaviour was done confidently or cautiously) (Figure 2). Similar trends were detected across resource points, although at carcasses, foxes spent a significantly higher amount of time on average in cautious locomotion than confident locomotion (*p* = 0.02) (Figure 2). Foxes foraging at rabbit warrens (i.e., hunting) were always observed in a confident state. Scent marking was also observed exclusively confidently. Overall, foxes were more likely to be detected in a confident, rather than cautious, behavioural state. Confident states at carcasses were engaged in more frequently while investigating (23 times more often), sniffing (8.5 times), locomoting (7.5), and foraging (5.5 times), while confident foraging was engaged in more frequently than cautious foraging at water sources (6.6 times) (Table 4).

Scent marking was most common at carcasses with a rate of one scent mark every 88.5 s, followed by water points at one scent mark every 92.6 s. Scent marking was observed much less frequently at rabbit warrens, with only one scent mark every 9.3 min. Fox scent marking comprised of scat deposition and urination (n = 32), raking (n = 1), and rubbing (n = 1).

**Figure 3:**
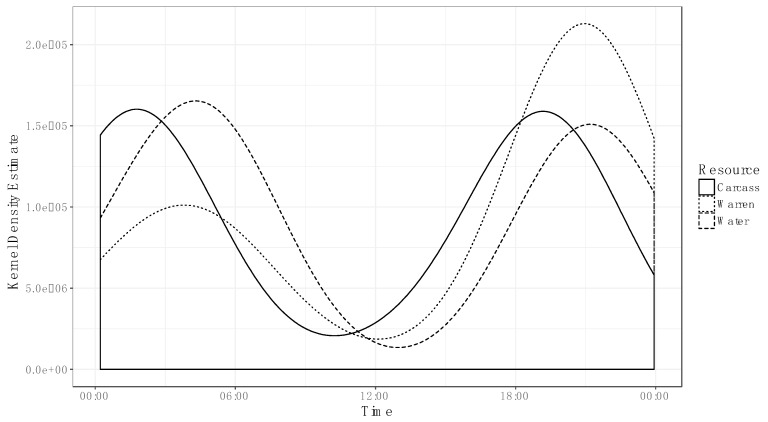
Fox temporal activity patterns at water points, rabbit warrens and carcasses gathered with camera traps in the Painted Desert, South Australia in the winters of 2016–2018. Solid line represents carcass temporal activity patterns, dashed line represents water points and dotted line represents rabbit warrens. Overlap coefficient between the three resource points is 56%.

Foxes primarily accessed resource points between dusk and dawn (06:00 and 18:00), but they visited each at slightly different times throughout the night, overlapping at 56% (Figure 3). Fox activity at carcasses was concentrated at two peaks, in the early morning (00:00–03:00) and evening (18:00–22:00). Similarly, activity at water sources was most frequent between 03:00 and 06:00 as well as 20:00 and 23:00. Rabbit warren activity was concentrated into a single peak in the evening (19:00–22:00).

Fox social behaviour comprised of two pairs at two carcasses, lasting in total for 5.10 minutes. Fox pairs spent the highest average proportion of their time sniffing (43%), followed by locomotion (33%) and foraging (27%). During this time, they played (n = 6), greeted one another (n = 5), and scent marked (n = 4).

## 4. Discussion

Foxes were surprisingly confident at resource points shared with territorial dingo packs, when free from human persecution. We had hypothesised that the threat presented by socially stable dingoes would induce foxes, more often than not, to be “on their toes” when visiting these peaks in the landscape of fear. On the whole, we found that foxes were much more likely to express their behaviours in confident states while at resource points, suggesting that foxes are not living in a state of fear. However, the evidence of cautious behaviours exhibited by foxes at resource points exemplifies the suppressive effects of apex predators, and mirrors behaviours observed in other fox populations coexisting with apex predators around the world [16,55].

The behaviour of foxes may be influenced by both their own social stability and that of their predators. Social stability in apex predators is a key driver of ecosystem function and has significant ecological flow-on effects [6]. The protection of predators and the promotion of their social stability enables coexistence between predators [56]. Cheetahs (*Acinonyx jubatus*) and spotted hyenas (*Crocuta crocuta*) living in the protected areas of Serengeti National Park, Tanzania, coexist with lions (*Panthera leo*) through “moment-to-moment” temporal avoidance of the apex predator, suggesting that smaller predators have a developed understanding of the spatiotemporal activities of lions, and how to behave in order to avoid them [57]. We propose that when a population of apex predators is socially stable, sympatric mesopredators may also be increasingly bold due to the territorial stability of apex predators, [41] potentially reducing the risk involved with spatially avoiding predators.

Although foxes were more likely to be confident at carcasses, when foxes were locomoting cautiously they did it significantly longer than when they did it confidently. This suggests that when foxes perceive increased risk at carcasses, they alter their behaviour to reduce the threat of encountering a dingo. Cattle carcasses are a valuable resource in arid ecosystems and dingoes regularly feed upon them [58]. The high value of carcasses to dingoes, and the increased caution that foxes exhibit on occasion, may suggest that dingoes are increasingly territorial and defensive of carcasses over other resource points. We acknowledge our video data of fox behaviour over our three-year sampling period, is limited and should be interpreted as such.

Evidence for this is emphasised by dingoes’ scent marking all of the carcasses surveyed, a behaviour that indicates ownership and territoriality in large canids [21,41]. In apparent ‘disregard’ for dingo territoriality, foxes regularly scent-marked resource points, including on large carcasses and water sources heavily marked and visited by dingoes. Similar observations of foxes marking existing apex predator scats have been observed in Poland, where foxes were observed inspecting scats of lynx and scent-marking over them [13]. Likewise, grey foxes (*Urocyon cinereoargenteus*) have been observed remarking the scent marks of pumas (*Puma concolor*) [59]. The functional benefits of re-marking scats of apex predators can only be speculated on, but it may serve to communicate to both conspecifics and predators. Further research is required to develop a deeper understanding of the role of over marking in the behavioural interactions involving apex- and meso-predators.

Observations of fox sociality in the wild are rare because foxes spend large amounts of time alone, however, we observed fox social behaviour at two carcasses. This may be attributed to our study being conducted in the winter, during their mating season [60]. Pair interactions were comprised of amicable play and greeting, suggesting the two were either paired or kin. Play behaviour between pairs at carcasses commonly frequented by dingoes provides further evidence that these foxes were generally at ease in this landscape.

The foxes in this study were most commonly observed at resource points between dusk and dawn, which is consistent with observations that foxes are nocturnal in their native range [35,36]. Temporal overlap between the three resource points was relatively low (56%), suggesting that foxes may engage in routines in which they access different resource points at different times of the day.

Considering that dingoes have been shown to have strong suppressive effects on foxes [6,11,15,19], why where these foxes much more likely to be confident than cautious around these shared resources? One possibility is that socially-stable apex predators are more predictable and therefore less frightening. Foxes may be able to identify, anticipate, and appropriately respond to the risk of dingo predation, therefore reducing the fear of unexpected attacks. Further research could help illuminate the role of social stability in shaping behavioural interactions between two of Australia’s most prominent predators.

## Figures and Tables

**Table 1:** Review of fox behaviour literature highlights the most common ecological contexts foxes are studied under.

Behavioural Study	Country	Wild/Captive	Foxes Killed	Apex Predators	References
Anti-predator behaviour	Australia	Wild	Yes	Dingo (*Canis dingo*)	[19]
	Canada	Wild	Unstated	Coyote (*C.latrans)*	[20]
	Croatia	Wild	No	Wolf *(C.lupus)*	[16]
	Israel	Wild	No	Golden jackal (*C.aureus*)	[12]
	North America	Wild	Unstated	Coyote	[9]
	Poland	Wild	Unstated	Lynx (*Lynx lynx*)	[13]
Fox kit ethogram	Switzerland	Wild	Unstated	Unstated	[21]
Reproduction	North America	Wild	Yes	Unstated	[22]
Sociality	England	Wild	Yes	No	[23]
	England	Captive	N/A	N/A	[24]
	North America	Captive	N/A	N/A	[25]
Scent marking	Canada	Wild	Unstated	Unstated	[26]
	Israel & North America	Both	Unstated	Unstated	[27]
	Spain	Wild	Unstated	Unstated	[28]
Spatiotemporal patterns	England	Wild	Yes	No	[29]
	England	Wild	Yes	No	[30]
	England	Wild	Yes	No	[31]
	England	Wild	Yes	No	[32]
	Italy	Wild	Yes	No	[33]
	Italy	Wild	Yes	Unstated	[34]
	Italy	Wild	Yes	Unstated	[35]
	Japan	Wild	Yes	Unstated	[36]
Feeding behaviours	Switzerland	Captive	N/A	N/A	[37]
	Sweden	Wild	Unstated	Unstated	[38]

Data gathered for this review comes from a Web of Science search, using “red fox behaviour” as the search term, refined for “behavioural sciences”. Reference trails were also included in the review. Unstated was noted if authors did not mention whether the variable in question was present during their study. N/A refers to a variable not being applicable to the study (e.g., Foxes killed is not relevant to a study conducted in captivity).

**Table 2:** Ethogram for foxes at resource points. Modifiers further describe the behaviour observed.

Behaviour State	Modifiers	Definition
Locomotion (S)	A) Walk/Run/Jump/Perch	All spatial movements
	B) Cautious/Confident	
Sniffing (S)	Cautious/Confident	Exploring area of interest leading with the
		nose, the head moves up and down with neck
		extended
Digging (S)	Cautious/Confident	Investigation of ground utilising the front two
		paws to remove a layer of soil
Vigilance (S)	Low/High	Examination of the surrounding environment
		in a state of alert or heightened awareness,
		the head moves directionally, head is moving
		rapidly or focused on an object or location.
		Individuals are positioned low to the ground
		with legs splayed (see Figure 1D)
Foraging (S)	A) Scavenging/Drinking/Hunting	The act of feeding on carrion, ingesting water
		from a natural or anthropogenic water source
		or hunting for prey
	B) Cautious/Confident	
	C) Alone/Social	
Scent marking (S)	A) Defecation/Raking/Rubbing	The raising of a hind leg or leaning into a
		squat position in order to deposit urine or
		scats onto a point of interest, using a paw to
		rake the ground or the act of rubbing face,
		paw or tail glands on an object
	B) Alone/Social	
Flight (P)	Startled Jump/Startled Flee	Dramatic and exaggerated responses to
		environmental or camera born stimuli. Body
		movements are rapid, legs, torso and head
		perform sudden and reckless movements to
		jump or flee away from the location where
		they were startled
Investigating (S)	Cautious/Confident	The act of surveying the environment.
		Head moves directionally, can be performed
		stationary or during locomotion
Frustration (P)		An outburst of frustration manifested by
		biting or gnawing on an object in the
		environment
Head shake (P)		A rapid shaking of the head in an attempt to
		remove or dislodge an item of irritation
Salivating (P)		A display of hunger at the anticipation of
		food involving the licking of the outside of
		an individual’s mouth
Resting (S)		An absence of discernible activity. An
		individual laying on the ground with all four
		legs relaxed or sitting down on back end with
		front paws fully extended, supporting the
		individual. Individual is motionless. Head
		may be focused on the ground, sky or on
		nothing in particular but is motionless
Greeting (S)	Facial/Posterior/Denial	The act of sniffing a conspecific to identify and
		communicate with the individual
Play (S)	Jump/Chase	One individual actively solicits a non-agonistic interaction, with random and exaggerated movements

Point events describe instantaneous behaviours (P). State events describe continuous behaviour (S).

**Table 3:** Descriptions of some red fox ethogram modifiers.

Behaviour	Modifiers	Description
Locomotion	A) Type	*Walk*: Slow quadrupedal movement *Run*: Fast quadrupedal movement *Jump*: Vertical or horizontal jump *Perch*: The lifting of two paws onto an object in order to investigate a resource or object of interest
	B) Attitudinal	*Confident*: Head not focused on anything in particular, head movements are relaxed, ears are relaxed and kept vertical (unless sound is heard, if so, ears will move directionally), little concern over movement. Tail held high, parallel to the ground, level with the back, may have a kink towards the end pointing upwards *Cautious*: Head moves erratically, ears pricked forward, cautious paw placement with back feet placed firmly with movement only occurring in front feet, stands with legs close together and bent. Tail positioned closer to the back legs, lower than level with the back, with no kink, shoulders are raised
Sniffing	Attitudinal	*Confident*: Sniffs are long and pronounced, little concern shown for anything apart from the object being sniffed. Head not focused on anything in particular, head movements are relaxed. Ears relaxed and kept vertical (unless sound is heard, if so, ears will move directionally), little concern over movement. Tail held high, parallel to the ground, level with the back, may have a kink towards the end pointing upwards *Cautious*: Sniffs are short. Head moves erratically, ears pricked forward, cautious paw placement with back feet placed firmly with movement only occurring in front feet stands with legs close together and bent. Tail positioned closer to the back legs, lower than level with the back, with no kink, shoulders are raised
Vigilance	Intensity	*Low*: Head is most commonly focused on a single location, can be represented by low to moderate speed head movements, neck is extended, stands with legs close together shoulders are raised. Can be performed standing or sitting quadrupedally. Tail is position is lower than the level the back *High*: Head raised and moves erratically and quickly, regularly change focal point, neck is heavily extended, ears are pricked forward, stands with legs close together and shoulders are raised. Can be performed standing or sitting quadrupedally. Tail positioned closer to the back legs, lower than level with the back, with no kink
Foraging	A) Type	*Scavenging*: The investigation of carrion resulting in an individual attempting to or successfully feeding *Drinking*: The act of utilising either an anthropogenic or natural water resource. *Hunting*: The act of actively searching for and/or consuming live prey
	B) Attitudinal	*Confident*: Individual attempting to consume resource makes slow movements, does not jump back after consuming the resource, consumes resource atop or very nearby resource. Ears perched vertical. Tail held high, parallel to the ground, level with the back, may have a kink towards the end pointing upwards *Cautious*: Individual attempting to consume resource is extremely jumpy, making erratic movements, ears perched forward, neck as elongated as possible to keep the majority of the body as far from resource as possible. Tail positioned closer to the back legs, lower than level with the back, with no kink, shoulders are raised. If possible, fox may take resource away from the resource to consume (most common during scavenging)
	C) Social	*Social*: The act of foraging with one or more conspecifics
Scent marking	Type	*Defecation*: The act of squatting or raising a hind leg in order to spray urine or deposit faeces in the environment *Raking*: The act of dragging or clawing the dirt with paws in order to transfer scent *Rubbing*: The act of rubbing facial or tail scent glands on objects of interest to transfer scent *Social*: Scent marking in a group with more than one conspecific
Flight	Type	*Startled jump*: Quick jump backwards, erratic and quick movement. Limbs move in unison, back is arched during the jump, fox will land behind the point it jumped from *Startled flee*: Commonly initiated through a quick turn in opposite direction the fox was previously facing, then engaging in very fast running away from a specific location. Foxes head and tail will move erratically during the behaviour
Investigating	Attitudinal	*Confident*: Body is relaxed, head movements are slow, ears are vertical, shoulders are lower, tail held high, parallel to the ground, level with the back, may have a kink towards the end pointing upwards *Cautious*: Head movements are slow, individual is not focused on a single point. Ears move directionally, shoulders are raised, tail is positioned towards the back legs
Greeting	Type	*Facial*: The act of sniffing the face and/or glands of the face of a conspecific *Posterior*: The act of sniffing the anus, anal glands or genitals of a conspecific *Denial*: The movement or jumping away from a conspecific after an attempted greeting
Play	Type	*Jump*: Leaping towards or away from a conspecific in a non-agonistic manner, with random and exaggerated movements *Chase*: The running or walking after or away from a conspecific in a non-agonistic manner, with random and exaggerated movements

**Table 4:** Descriptive statistics of the time allocated to different behavioural states at the three resource points, depending upon whether the behaviours were expressed cautiously or confidently.

		Cautious			Confident		
Resource	Behaviour	No.	Total Time	Average Time	No.	Total Time	Average Time
Carcass	Digging		0	N/A	7	10.5	1.5
	Foraging	8	80.3	10.0	44	386.6	8.8
	Investigating	1	8.0	8.0	23	112.7	4.9
	Locomotion	8	66.4	8.3	60	191.8	3.2
	Scent marking		0	N/A	13	16.2	1.2
	Sniffing	6	7.0	1.2	51	218.0	4.3
	Vigilance	30	108.3	3.6		0	N/A
Warren	Foraging		0	N/A	9	65.2	7.2
	Investigating	18	98.2	5.4	34	183.7	5.4
	Locomotion	26	89.3	3.4	67	160.2	2.4
	Scent marking		0	N/A	2	2.1	1.0
	Sniffing	18	54.7	3.0	32	93.2	2.9
	Vigilance	28	101.7	3.6		0	N/A
Water	Digging		0	N/A	2	0	0
	Foraging	5	47.5	9.5	33	362.3	11.0
	Investigating	15	48.6	3.2	10	46.5	4.7
	Locomotion	42	131.1	3.1	99	316.0	3.2
	Scent marking		0	N/A	16	25.0	1.6
	Sniffing	15	59.3	4.0	41	145.0	3.5
	Vigilance	55	236.7	4.3		0	N/A
Total		275	1137.1	4.1	543	2335.1	4.3

Time is represented in seconds (s).

## Supplementary Materials

The following are available online at https://www.mdpi.com/2076-2615/9/11/907/s1, Table S1: Negative binomial model results of the proportion of time spent in each behaviour at different resource points.
