# Supplementary material for: The Wily and Courageous Red Fox: Behavioural Analysis of a Mesopredator at Resource Points Shared by an Apex Predator
Source: Animals (Basel). 2019 Nov 1;9(11):907. doi: 10.3390/ani9110907 (PMC6912404; doi:10.3390/ani9110907)
Supplement: Supplementary file 1 [file animals-09-00907-s001.pdf]

Supplementary material:

Table S1. Negative binomial model results of the proportion of time spent in each behaviour at different resource points.

	Omnibus test results			Post-hoc test results	
**Behaviour**	**Chi-squared**	**df**	***p***	**Resource**	**95% CIs**
**Locomotion**	0.8941	2	0.6395	Water	0.292–0.550
				Warren	0.335–0.686
				Carcass	0.300–0.705
**Foraging**	0.6477	1	0.7234	Water	0.430–1.096
				Warren	0.371–0.889
				Carcass	0.163–1.543
**Investigating**	0.144	2	0.931	Water	0.179–0.833
				Warren	0.262–0.716
				Carcass	0.175–0.483
**Sniffing**	0.879	2	0.644	Water	0.351–0.835
				Warren	0.247–0.702
				Carcass	0.296–0.744
**Digging**	0.256	1	0.613	Water	0.026–5.03
				Carcass	0.019–1.32
**Scent marking**	0.209	2	0.900	Water	0.037–0.715
				Warren	0.0001–44.705
				Carcass	0.016–0.811
**Vigilance**	0.280	2	0.869	Water	0.174–0.553
				Warren	0.132–0.864
				Carcass	0.103–0.595

Given that there was no significance difference between groups, we report only the upper and lower confidence intervals.
